# Characteristics and effectiveness of diabetes self-management educational programs targeted to racial/ethnic minority groups: a systematic review, meta-analysis and meta-regression

**DOI:** 10.1186/1472-6823-14-60

**Published:** 2014-07-19

**Authors:** Ignacio Ricci-Cabello, Isabel Ruiz-Pérez, Antonio Rojas-García, Guadalupe Pastor, Miguel Rodríguez-Barranco, Daniela C Gonçalves

**Affiliations:** 1 Nuffield Department of Primary Care Health Sciences, University of Oxford, New Radcliffe House, 2nd floor, Walton Street, Jericho OX2 6NW, UK; 2 CIBER en Epidemiologia y Salud Pública (CIBERESP), Barcelona, Spain; 3 Andalusian School of Public Health, Granada, Spain

**Keywords:** Diabetes type 2, Self-management, Educational interventions, Systematic literature review, Meta-analysis, Meta-regression

## Abstract

**Background:**

It is not clear to what extent educational programs aimed at promoting diabetes self-management in ethnic minority groups are effective. The aim of this work was to systematically review the effectiveness of educational programs to promote the self-management of racial/ethnic minority groups with type 2 diabetes, and to identify programs’ characteristics associated with greater success.

**Methods:**

We undertook a systematic literature review. Specific searches were designed and implemented for Medline, EMBASE, CINAHL, ISI Web of Knowledge, Scirus, Current Contents and nine additional sources (from inception to October 2012). We included experimental and quasi-experimental studies assessing the impact of educational programs targeted to racial/ethnic minority groups with type 2 diabetes. We only included interventions conducted in countries members of the OECD. Two reviewers independently screened citations. Structured forms were used to extract information on intervention characteristics, effectiveness, and cost-effectiveness. When possible, we conducted random-effects meta-analyses using standardized mean differences to obtain aggregate estimates of effect size with 95% confidence intervals. Two reviewers independently extracted all the information and critically appraised the studies.

**Results:**

We identified thirty-seven studies reporting on thirty-nine educational programs. Most of them were conducted in the US, with African American or Latino participants. Most programs obtained some benefits over standard care in improving diabetes knowledge, self-management behaviors and clinical outcomes. A meta-analysis of 20 randomized controlled trials (3,094 patients) indicated that the programs produced a reduction in glycated hemoglobin of -0.31% (95% CI -0.48% to -0.14%). Diabetes knowledge and self-management measures were too heterogeneous to pool. Meta-regressions showed larger reduction in glycated hemoglobin in individual and face to face delivered interventions, as well as in those involving peer educators, including cognitive reframing techniques, and a lower number of teaching methods. The long-term effects remain unknown and cost-effectiveness was rarely estimated.

**Conclusions:**

Diabetes self-management educational programs targeted to racial/ethnic minority groups can produce a positive effect on diabetes knowledge and on self-management behavior, ultimately improving glycemic control. Future programs should take into account the key characteristics identified in this review.

## Background

The prevalence of type 2 diabetes rapidly rose over the past three decades. However its burden is not homogeneously distributed: racial and ethnic minorities usually experience higher prevalence than their non-minority counterparts [1] and are at higher risk of developing diabetes-related complications such as blindness, kidney damage, or depression, impacting both quality of life and mortality rates [2,3].

Self-management, defined as the patient’s ability to manage not only the symptoms inherent to a chronic condition but also its treatment and associated lifestyle changes [4], has become increasingly important in the treatment of type 2 diabetes [5,6]. However, because adequate diabetes self-management (DSM) may require considerable lifestyle changes to several domains, namely having a healthy diet, exercising, or glucose monitoring, not all the patients are able to properly follow the self-management plans agreed with their healthcare professionals or advised by clinical guidelines. Racial and ethnic minorities are less likely to engage in DSM behaviors than other population groups, which partially explains observed disparities in health outcomes [7]. Some of the barriers faced by these groups for achieving an adequate DSM are related to characteristics of the groups (such as health literacy or health beliefs) and also of the healthcare system (namely accessibility of culturally sensitive information) [8,9].

Several review studies have assessed the effect of DSM educational programs on the general population [6,10-17]. Those studies have established that DSM educational programs can improve glycemic control [11-16], and identified the key characteristics for improving glycemic control, including face-to-face delivery, teaching methods based on cognitive reframing [11], and higher contact time between participant and educator [16]. A smaller number of studies have reviewed the evidence of the effect of these programs on racial/ethnic minorities [18-20]. Only one study [18] examined their impact on glycemic control, observing a reduction on glycated hemoglobin of 0.32%. There were however some limitations underlying that study, as some of the included interventions combined educational programs with other types of quality improvement strategies, making it difficult to disentangle the individual effect of the educational components. More importantly, no previous meta-regression study has identified the key common characteristics of successful educational programs targeted to racial/ethnic minority groups. This represents a considerable gap in the literature, as programs for racial/ethnic minority groups should have different components from those targeting other population groups, namely addressing the cultural idiosyncrasies associated with each group. Therefore it is not reasonable to assume that the same type of successful program will be equally successful when applied to racial/ethnic minority groups. Additionally, in the past years several clinical trials examining the impact of educational programs to improve diabetes self-management on racial/ethnic minorities have been published, making now possible a more detailed review and analysis of the available evidence regarding the effectiveness of these interventions.

In this work we systematically reviewed DSM educational interventions specifically targeted to racial/ethnic minority groups. We studied the characteristics and costs of the interventions, and analyzed their impact on diabetes knowledge, self-management behaviors, and clinical outcomes. Whenever data were available, we performed meta-analyses to examine the short and long-term effects of the interventions, and meta-regressions to identify common characteristics of the interventions associated with better results.

## Methods

The review and its procedures were planned, conducted, and reported according to the Preferred Reporting Items for Systematic Reviews and Meta-Analyses (PRISMA) guidelines [21].

### Data sources and searches

A comprehensive core search strategy was developed for Medline through Ovid (combining MeSH terms and keywords) and then adapted and implemented in EMBASE and CINAHL (search strategy available in Additional file 1: Table S1). Gray literature and additional articles were searched in twelve more bibliographic sources (Additional file 2: Table S2). The search was not restricted by language or publication date. For all the references selected to be included in the review, backward and forward citation searches were performed in ISI Web of Knowledge. All searches were conducted in October 2012. A bibliographical database was created using EndNote X6, and used to store and manage the retrieved references.

### Study selection

We included studies analyzing the effectiveness of DSM educational programs targeted to racial/ethnic minority groups with type 2 diabetes. We only included those studies in which at least 90% of the participants pertained to a racial/ethnic minority group considered to be at a higher risk for diabetes complications than the majority population group. Racial/ethnic minority group was defined as a population group with a race or ethnicity different from that of the majority population group of the host country. Groups at higher risk of diabetes complications were identified based on available literature. Interventions had to be exclusively educational, without including any other component such us financial incentives, clinician education or case management. In order to avoid possible comparisons between programs carried out in very heterogeneous settings, with very different health systems and population needs, we restricted this review to those interventions conducted in countries that were members of the OECD [22], when study selection was conducted (November 2012).

Eligible designs were randomized controlled trials (RCTs), including cluster randomized controlled trials; controlled trials, including quasi-randomized trials; controlled before-after studies; and non-controlled before-after studies. Studies including a control group were only eligible in case the intervention was compared with care as usual.

Titles and abstracts were screened for eligibility, and those fulfilling the inclusion criteria were included in the next stage, where the full texts of the selected articles were retrieved and assessed. Those that met the inclusion criteria were included for data extraction. Two reviewers independently screened citations, and any disagreements were solved by consensus with a third reviewer.

### Data extraction and quality assessment

We designed and used structured forms to extract information of interventions’ characteristics and their effectiveness. We used a previously developed taxonomy of DSM educational programs to characterize the interventions [23]. The following information was extracted: setting, ethnic group, administration formats, teaching methods, educational contents, educators’ background, use of peer educators, and duration. Information of interventions’ cost and cost-effectiveness was also extracted.

We critically appraised the studies using the Quality Assessment Tool for Quantitative Studies [24], which enables the assessment of both internal and external validity, classifying them into three categories (good, fair or poor) depending on six aspects: selection bias, study design, confounders, blinding, data collection and withdrawals/ dropouts. Two reviewers independently extracted all the information and critically appraised the studies. Disagreements were solved through discussion with a third reviewer. When necessary we contacted the authors of the studies to request additional information.

### Data synthesis and analysis

The effectiveness of the interventions was assessed in terms of their impact on 1) diabetes knowledge, 2) diabetes self-management behavior, and 3) clinical outcomes. Diabetes knowledge was ascertained by measures reflecting the theoretical knowledge the patients had about their condition. Diabetes self-management behavior measured the performance of specific activities related to adequate DSM (diet, exercise, glucose control, foot self-examination, etc.). Clinical outcomes included hemoglobin A1c (HbA1c), body mass index (BMI), or blood pressure, amongst others. All outcomes in all the studies were examined and classified as measuring one of these three domains. Variables that measured other domains were not included in the analysis.

Additionally, we conducted independent meta-analyses to analyze short and long-term (six months post-intervention) effect of the interventions on glycemic control. Eligibility criteria for the meta-analyses included randomized controlled trials comparing the interventions with usual care. The mean (standard deviation) of HbA1c levels in each study were extracted. This information was transformed into weighted mean difference and 95% confidence intervals (CI) were calculated and combined using random-effects models. We imputed unreported standard deviations by use of established methods [25]. Heterogeneity was quantified by the I^2^ statistic, where I^2^ ≥ 50% was considered evidence of substantial heterogeneity [26]. Sources of heterogeneity were investigated by a Galbraith plot. Publication bias was quantitatively assessed with Egger test.

We used bivariate meta-regression to explore relationships between effect size (ES) and interventions characteristics. The number of included studies was insufficient to perform a multivariate regression analysis. We conducted a sensitivity analysis, excluding the studies with higher risk of bias. All analyses were conducted with Stata, version 12.0 (StataCorp, College Station, Texas). For all the analyses, statistical significance was accepted at *p* < 0.05.

## Results

### Article identification

**Figure 1:**
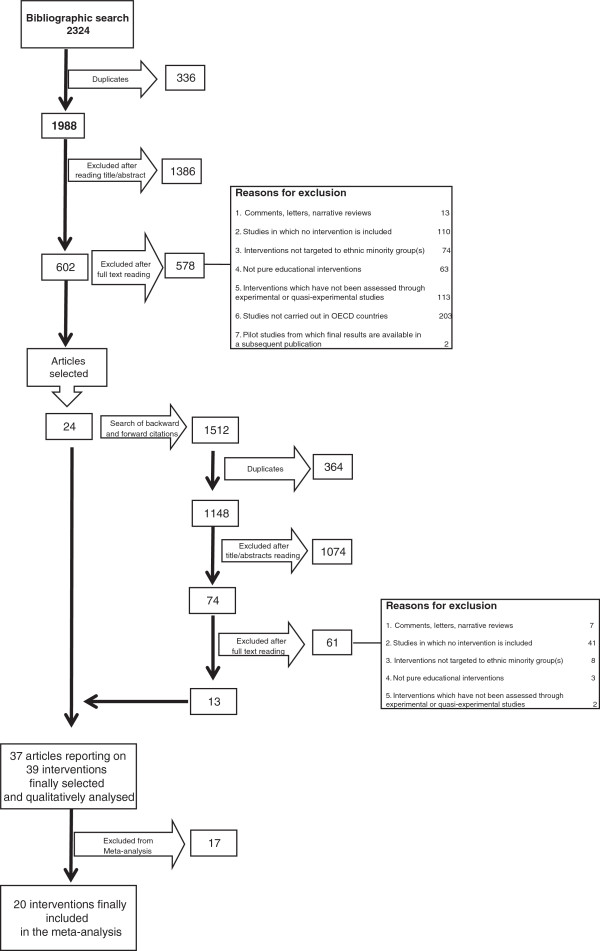
Summary of evidence search and selection.

Figure 1 reports the screening process. A total of 1,988 unique references were retrieved. 1,386 references were excluded based on title and abstract, resulting in 602 references being included in the next stage. Full text articles were retrieved and assessed, with 24 studies meeting the eligibility criteria. Backward and forward search of these 24 articles identified thirteen additional studies, resulting in thirty seven articles being included in the review [27-63]. Thirty five of them reported one single intervention, whereas two articles reported two distinct interventions per article. Overall, thirty seven articles were identified, which analyzed the effectiveness of thirty nine different interventions.

### Characteristics of the studies and interventions

Table 1 shows the aggregated characteristics of the interventions and of the studies used to assess their effectiveness. Most of the studies identified were conducted in the US (92%), and published from 2001 onwards (84%). Almost two thirds of the studies were RCTs (73%), and approximately two thirds presented moderate or low risk of bias (65%).

**Table 1:** Characteristics of the studies and interventions

		Number (N)*	**Percentage (%)**
**Characteristics of the studies (n = 37)**			
Country where the intervention took place			
	United States	34	92
	United Kingdom	2	5
	Netherland	1	3
Design			
	Randomized controlled trial	27	73
	Quasi-experimental	10	27
Years of publication			
	Before 2001	6	16
	2001-2006	16	43
	2007-2012	15	41
Risk of bias			
	Low	8	22
	Moderate	16	43
	High	13	35
Number of participants		159 (117)†	7-529‡
**Characteristics of the interventions (n = 39)**			
Ethnicity			
	African American	13	33
	Latinos	16	41
	Asiatic	2	5
	Alaskan-Eskimos	1	2
	Multiethnic group	7	18
Place where intervention took place			
	General Practice	16	44
	Community Center	12	33
	Home	3	8
	Hospital	1	3
	Other	4	11
Setting			
	One-on-one	13	33
	Group	17	44
	Both	9	23
Family invited to take part in the intervention			
	Yes	12	31
	No	27	69
Delivery			
	Face-to-face	27	69
	Telecommunication	5	13
	Both	7	18
Number of teaching methods			
	1	7	18
	2	24	62
	3 or more	8	26
Teaching method*			
	Didactic	32	82
	Goal-setting, dictated	8	21
	Goal-setting, negotiated	10	26
	Situational problem solving	23	59
	Cognitive reframing	7	18
	Other	2	5
Number of educational contents			
	One	7	18
	Two	5	13
	Three	5	13
	Four of more	22	56
Educational Content			
	Diet	33	85
	Exercise	24	65
	Self-monitored blood glucose	23	59
	Basic diabetes knowledge	21	54
	Medication adherence	11	28
	Psycho-social	19	49
	Other	11	28
Number of educators			
	One	18	46
	Two	14	36
	Three or more	4	10
Educators			
	Nurse	15	39
	Dietitian	21	54
	Psychologist	2	5
	Physician	2	5
	Research team or staff	4	10
	Other	16	41
	Unclear/unspecified	2	5
Peer provider			
	Yes	12	31
	No	26	67
Culturally adapted			
	Yes	33	85
	No	4	10
	Unclear/unspecified	2	5
Length of the interventions (months)		8.2 (8.4)†	0.25-48‡
Length of the interventions (number of sessions)		13.1 (13.6)†	1-52‡
Length of the sessions (minutes)		90 (52.9)†	14-240‡
Length of the interventions (total hours of intervention)		23.3 (36.7)†	0.25-180‡
Intensity (total hours of intervention per month)		4.9 (6.9)†	0.2-36‡

*N is not equal to 37 for all variables, as some characteristics were not reported for all the interventions. †mean (sd); ‡min-max.

In relation to the characteristics of the interventions, most of them targeted African American (33%) or Latinos (41%) and took place either in General Practices (41%) or in Community Centers (31%). Face to face was the most common format of delivery (70% of the interventions). Almost half of the interventions followed a group format (44%), whereas one third was delivered individually. Patients were encouraged to bring their relatives in nearly a third of the interventions (31%). On average programs lasted eight months and included 13 sessions, with each session lasting 90 minutes. Most of the programs included multiple teaching methods and multiple contents. Approximately half of the programs were delivered by multidisciplinary educators (54%).

### Effectiveness of the interventions

Additional file 3'; Table S3 shows the characteristics of each study and their impact on diabetes knowledge, self-management behaviors and clinical outcomes. Fifteen studies analyzed the impact of the interventions on diabetes knowledge [27,29,32,34,37-39,44,47,48,50,53,56,60,62]. In the majority of studies (nine out of fifteen), diabetes knowledge was only measured immediately after the intervention program was finished. Different types of instruments were used to measure outcomes such as diabetes knowledge and its complications or how to conduct adequate diabetes self-management. Eleven of these studies observed that the interventions significantly improved patients’ knowledge, whereas the remaining four did not observe significant effects. Health beliefs were additionally analyzed in two studies, without observing a significant improvement.

Twenty studies examined the potential of the interventions to improve self-management behaviors [27,28,30-32,34-36,43-45,50,54,55,57-62]. Behavioral outcomes were heterogeneous, being related in most instances to dietary or physical activity behaviors, but also to behaviors related to blood glucose testing, foot care, or medication adherence. Fifteen out of twenty studies (75%) observed that the interventions produced improvements in behavioral outcomes. Interventions were more successful in improving dietary behaviors than in promoting physical activity or medication adherence.

Thirty-one studies assessed the impact of the interventions on clinical outcomes [27,29-33,37-44,46-60,62,63]. The most frequent clinical outcome was HbA1c, but blood pressure, fasting blood glucose or BMI were also included in a substantial number of studies. Twenty two studies (71%) observed that the educational programs produced statistically significant improvements in clinical outcomes. Educational programs more frequently improved fasting blood glucose, HbA1c and blood pressure (improved in 71%, 59%, and 57% of the studies, respectively) than other clinical outcomes such as lipid profile (40%), weight/BMI (28%) or waist circumference (25%).

Costs were reported in only two studies [33,40]. The cost per patient per year was approximately $280 in the intervention developed by Banister et al. [33] and $461 in the intervention by Culica et al. [40]. No study included a formal cost-effectiveness analysis.

### Effectiveness of the intervention on glycated hemoglobin

**Figure 2:**
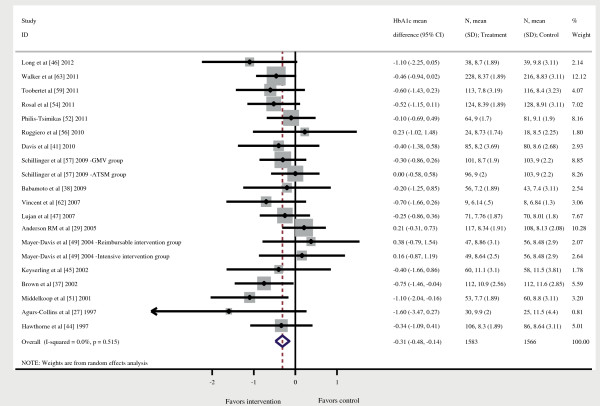
**Forest plot.** HbA1c, Glycated hemoglobin; 95% CI = 95% confidence interval.

Twenty-one interventions employed HbA1c measures and were included in an initial meta-analysis that assessed possible baseline HbA1c differences between intervention and control groups. No statistically significant differences were found (HbA1c mean difference = -0.02% [95% CI -0.22% to 0.18%]). A second meta-analysis was conducted to estimate the pooled difference in HbA1c between the intervention and control group immediately after the intervention was completed, observing a significant reduction in the overall HbA1c of -0.47% (95% CI -0.76% to -0.17%). Although heterogeneity was high (I^2^ = 66.3%), it was mainly associated with one intervention [55], and once that intervention was excluded, heterogeneity was reduced to 0%. Twenty interventions were therefore included in the final meta-analysis, reporting on 3,094 patients (1,551 in the intervention and 1,543 in the control group). The combined effect of the intervention produced a significant reduction in the overall HbA1c of -0.31% (95% CI -0.48% to -0.14%) (Figure 2). Egger test indicated the absence of publication bias (*p* = 0.22). One of the studies included in the meta-analysis presented high risk of bias [44]. We conducted a sensitivity analysis excluding it, obtaining very similar results.

Only three studies measured HbA1c at six months post-intervention [30,45,52]. A meta-analysis of these three studies observed a reduction in pooled HbA1c of -0.47%, although no significant differences were observed (*p* = 0.13).

### Intervention characteristics associated with treatment effects

We conducted bivariate meta-regressions of the 20 RCTs included in our meta-analysis in order to identify the characteristics associated with increased short-term HbA1c reduction (results reported in Table 2). Interventions delivered face to face obtained better results than those interventions supported by telecommunication. Also, those delivered individually performed better than those delivered in a group format. As for the teaching methods, both interventions that employed cognitive reframing techniques, as well as those including only one teaching method, were associated with better outcomes. Finally, those interventions that included at least one peer educator produced significantly better effects than those not including any peer educator.

**Table 2:** Meta-regression of the effect of intervention´s characteristics on pooled glycated hemoglobin (N = 20)

				**Number interventions**^**a**^	**SMD**	**95% CI**	**Residual I**^**2**^
Country							0.00%
		US		18	-0.28**	-0.47; -0.09	
		Netherland		1	-0.82	-1.85; 0.21	
		UK		1	-0.06	-0.88; 0.77	
Year							1.74%
		Prior 2001		2	-0.51	-1.29; 0.26	
		2001-2006		6	0.32	-0.52; 1.15	
		2007-2012		12	0.18	-0.60; 0.97	
Setting							0.00%
		Primary Care		9	-0.23	-0.58; 0.11	
		Community center		6	-0.03	-0.48; 0.41	
		Other		5	-0.24	-0.74; 0.26	
Mean HbA1c at baseline in intervention group							0.08%
		HbA1c < 9%		10	-0.39**	-0.96; 0.17	
		HbA1c ≥ 9%		10	0.06	-0.32; 0.43	
Target population							3.33%
		Ethnic minority		10	-0.30	-0.58; -0.04	
		Rural ethnic minority		3	0.31	-0.41; 1.03	
		Women from ethnic minority group		2	-0.23	-1.04; 0.58	
		Other (elderly or low income or low literacy)		5	-0.05	-0.47; 0.36	
Ethnic minority group							0.00%
		African-American		7	-0.10	-0.48; 0.28	
		Latinos		7	-0.31	-0.80; 0.17	
		Asiatic		2	-0.54	-1.28; 0.20	
		Multi-ethnic		4	-0.15	-0.66; 0.35	
Individual Vs Group delivered							0.00%
		Individual		7	-0.45	-0.75; -0.14	
		Group		7	0.13	-0.29; 0.56	
		Both		4	0.32	-0.14; 0.79	
Patients accompanied by family							0.00%
		No		5	-0.23**	-0.44; -0.04	
		Yes		15	-0.38	-0.84; 0.07	
Face to face Vs. telecommunication							0.00%
		Exclusively face to face		12	-0.37**	-0.62; -0.12	
		Exclusively telecommunication		3	-0.08	-0.53; 0.37	
		Combining face to face and telecommunication		5	0.33	-0.11; 0.77	
Teaching methods							0.00%
		Didactic					0.39%
			Yes	14	-0.30**	-0.53; -0.07	
			No	6	-0.03	-0.42; 0.35	
		Goal setting negotiated					0.00%
			Yes	8	-0.29**	-0.57; -0.01	
			No	12	-0.03	-0.41; 0.34	
		Goal setting dictated					0.00%
			Yes	7	-0.26	-0.55; 0.02	
			No	13	-0.09	-0.48; 0.29	
		Situational problem solving					0.00%
			Yes	10	-0.28**	-0.50; -0.06	
			No	10	-0.08	-0.47; 0.30	
		Cognitive reframing					0.00%
			Yes	4	-0.47**	-0.91; -0.03	
			No	16	0.20	-0.29; 0.68	
		Number of teaching methods used					0.00%
			1	4	-0.58**	-1.04; -0.12	
			2	10	0.37	-0.16; 0.92	
			3 or more	4	0.27	-0.27; 0.80	
Content							
		Diet					0.00%
			Yes	18	-0.35*	-0.54; -0.15	
			No	1	-0.74	-2.00; 0.50	
		Exercise					0.00%
			Yes	15	-0.33*	-0.54; -0.12	
			No	4	-0.20	-0.68; 0.28	
		Blood glucose self-monitoring					0.00%
			Yes	13	-0.26*	-0.48; -0.03	
			No	6	-0.39	-0.81; 0.04	
		Basic diabetes knowledge					0.00%
			Yes	8	-0.28*	-0.53; -0.02	
			No	11	-0.21	-0.60; 0.17	
		Medication adherence					0.00%
			Yes	9	-0.25*	-0.49; -0.05	
			No	10	-0.19	-0.58; 0.20	
		Psycho-social					0.00%
			Yes	11	-0.18*	-0.40; 0.03	
			No	9	-0.38	-0.76; 0.01	
	Number of contents						5.17%
		1 or 2		4	-0.36	-0.84; 0.12	
		3 or 4		9	0.02	-0.55; 0.58	
		5 or more		7	0.84	-0.48; 0.65	
Educators							
		Nurse					2.59%
			Yes	5	-0.02	-0.43; 0.39	
			No	14	-0.29*	-0.52; -0.06	
		Dietician					0.77%
			Yes	11	-0.10	-0.27; 0.47	
			No	8	-0.35*	-0.62; -0.08	
		Psychologist					2.47%
			Yes	1	0.05	-0.65; 0.76	
			No	18	-0.30*	-0.50; -0.11	
		Physician					0.00%
			Yes	2	0.21	-0.29; 0.72	
			No	17	-0.33*	-0.54; -0.13	
		Research team					2.13%
			Yes	2	-0.10	-0.77; 0.58	
			No	17	-0.29*	-0.49; -0.09	
Number of types of educators							0.00%
		One		11	-0.30*	-0.55; -0.04	
		Two		6	-0.41	-0.90; 0.07	
		Three or more		2	0.17	-0.34; 0.70	
Peer provider							0.00%
		Yes		7	-0.54	-0.93; -0.15*	
		No		13	0.29	-0.15; 0.74	
Duration of the intervention (months)				8.4 (1.4)†	-0.02	-0.05; 0.02	
Number of sessions				12.1 (2.1)†	-0.01	-0.02; 0.01	0.00%
Average duration of each session (hours)				1.5 (0.2)†	-0.01	-0.25; 0.23	0.00%
Total hours of intervention				21.9 (7.0)†	-0.01	-0.01; 0.01	0.00%
Intensity (number of hours/month)				4.6 (1.3)†	-0.01	-0.09; 0.07	1.60%

SMD = standardized mean difference; I^2^ = Variation in standardized mean difference attributable to heterogeneity; 95% CI = 95% confidence interval; HbA1c = glycated hemoglobin.

^a^N is not equal to 20 for all variables, as some characteristics were not reported for all the interventions.

*p < 0.05; **p < 0.001, † = mean (SE).

No statistically significant differences were observed for the total duration of the intervention, the number of sessions included, the duration of each session, the total number of hours of intervention or its intensity (number of hours per month).

## Discussion

In this systematic review we identified and characterized 39 DSM educational programs specifically targeted to racial/ethnic minority groups. Most programs produced some benefits over care as usual in improving diabetes knowledge, self-management behaviors, and clinical outcomes. Furthermore, meta-analyses indicated that these interventions decreased HbA1c, which was significant both from statistical and clinical perspectives. Larger reductions in HbA1c were observed in those interventions delivered individually and face to face, involving peer educators, based on cognitive reframing techniques, and employing a lower number of teaching methods. Long-term effects and cost-effectiveness were rarely assessed.

The estimated 0.31% reduction in HbA1c observed in our meta-analysis is modest but clinically significant, as evidence suggests that every percentage point decrease in HbA1c over 10 years is associated with a risk reduction of 21% for deaths related to diabetes, 14% for myocardial infarctions, and 37% for microvascular complications [64,65]. A substantial body of evidence for the effectiveness of educational interventions to improve glycemic control in general population has been generated [11,12,15,16], observing similar effects to the one obtained by our meta-analysis for racial/ethnic minority groups. Our results also reiterate those obtained in a previous meta-analysis of interventions targeting racial/ethnic minority groups (-0.32%) [18].

This is the first meta-regression study analyzing the effect of specific characteristics of educational programs targeted to racial/ethnic minorities. Moreover, meta-regressions of programs targeted to non-minority groups have explored a more reduced number of characteristics [11,16].

Educational programs delivered face to face produced a greater improvement in glycemic control than those delivered using telecommunication based formats. The comparative effectiveness of these two formats of administration is currently a topic of substantial interest, and there is no previous evidence in the context of self-management education in ethnic minorities. Although telecommunication programs have the potential to improve attrition rates, as they can help to overcome barriers such as competing responsibilities and distance to the service, they can represent an additional barrier to patients from racial/ethnic minority groups, who are more likely to have decreased access to information technologies and lower digital literacy [66].

Additionally, our meta-regression suggested that interventions delivered individually produced better results than those delivered in a group format. Previous research on general population has specifically explored this issue, without observing differences between individual and group administration [10,64]. Both the lower maintenance costs and the potential for promoting patient-patient interactions [67] make group-based interventions very appealing. Individual education, however, can more efficiently address patients’ individual characteristics and needs, producing better patient engagement. More research is needed to confirm our results.

Most of the educational programs included in our review were based on traditional didactic methods, either alone or in tandem with other educational techniques. However, those interventions based on cognitive reframing techniques produced better results. Similar results were obtained in a previous meta-regression of educational programs in the general population [11]. Furthermore, they corroborate previous findings that knowledge of lifestyle guidelines is a necessary but not the only factor required to facilitate the appropriate behavioral changes [15,17], suggesting that a patient’s inability to adhere to an adequate self-management might be grounded in motivational factors. The importance of motivational factors to promote the adherence to lifestyle interventions has been previously acknowledged and included in interventions targeting racial and ethnic minorities [68,69].

The involvement of peer providers also produced better results. The benefit of including peer educators has been previously suggested [70,71], and partially explained by the fact that peers can provide a more credible source of information, empower those involved, and reinforce learning through ongoing contact [72].

In order to estimate the complexity of the interventions, we calculated the number of teaching methods and educational contents included in each program. Contrary to our expectations, we observed an inverse association with HbA1c, indicating that less complex interventions led to greater improvements in glycemic control. This is the first study analyzing the relation between complexity and effectiveness, and more research is needed to confirm this potential dilution effect.

### Strengths and weaknesses

The main strength of this study is the comprehensiveness of the searches. Systematic and manual searches were performed in the most relevant bibliographic databases for biomedical research, as well as in specific sites of gray literature. We also examined the effect of a high number of intervention characteristics with the potential to produce better effects, some of which has not previously explored, namely the complexity of the programs or its intensity. Additional strengths are that we specifically focused our review on exclusively educational interventions (i.e., excluding those interventions with additional components such as case management, financial incentives or health provider education) and included sensitivity analysis excluding those studies with higher risk of bias.

Our review also has some limitations. First, although we attempted to identify studies conducted in OECD countries, a vast majority of the interventions were conducted in the US, which limits the external validity of our results. Second, our meta-analysis and meta-regression was restricted to glycemic control. Although we attempted to conduct meta-analyses on other relevant outcomes such as diabetes knowledge, they were not consistently available or uniformly measured. Finally, although formal tests on publication bias seemed to exclude its presence, we cannot completely rule out its existence.

### Remaining gaps in knowledge

More than 90% of the studies included in this review were conducted in the US, which limits the external validity of our results. Ethnic/racial inequalities in rates of diabetes-related complication have been observed in multiple countries and ethnic minorities [3]. Therefore, the effectiveness of interventions specifically targeting minorities needs to be assessed. This review also found that there is a considerable knowledge gap regarding the long-term effects of these interventions. Only about a fourth of the studies included had a post-test assessment, the majority of occurred within six months after the intervention ended. Given that type 2 diabetes is a chronic condition, it is crucial to understand not only that self-management educational programs can produce a discrete impact, but also whether the impact is sustained in the long term. Also importantly, a quarter of the interventions included in this review were evaluated through quasi-experimental studies. Some of these studies did not include a randomization element in the design, whereas other did not include a control group (non-controlled before-after studies). Moreover, a significant proportion of the studies (35%) presented a high risk of bias, which included small sample sizes, relevant confounders not adequately being controlled for, and participants not blinded to the intervention. Notwithstanding the difficulties underlying the execution of this type of complex clinical trials, larger and methodologically more robust trials are very much needed to confirm the findings of the present review, and to further identify characteristics of successful programs. Finally, only a small proportion of studies included cost-effectiveness estimation, which constitutes another important area for future research.

## Conclusions

In this systematic review we identified and analyzed DSM educational programs specifically targeted to racial/ethnic minority groups, observing that most of them can improve diabetes knowledge, self-management behavior, and clinical outcomes. Interventions producing higher improvements in glycemic control are those delivered individually and face to face, involving peer educators, based on cognitive reframing techniques, and employing a lower number of teaching methods. The long-term effects on patient-centered and clinically important outcomes, as well as cost effectiveness, remain unknown.

## Competing interests

The authors declare that they have no competing interests.

## Authors’ contribution

IRC and IRP designed the study. DCG, ARG and GP selected the articles and extracted relevant data. MRB conducted the statistical analysis. IRC drafted article. All authors provided input during the preparation of the manuscript, and approved the final version. IRC is the guarantor of this article.

## Pre-publication history

The pre-publication history for this paper can be accessed here:

http://www.biomedcentral.com/1472-6823/14/60/prepub

## Supplementary Material

Additional file 1: Table S1 Search strategy in Medline (Ovid). Click here for file

Additional file 2: Table S2 Registry of the Bibliographic Searches. Click here for file

Additional file 3: Table S3 Characteristics and Effectiveness of the Diabetes Self-management Educational Interventions. Click here for file
