# Supplementary material for: Characteristics and effectiveness of diabetes self-management educational programs targeted to racial/ethnic minority groups: a systematic review, meta-analysis and meta-regression
Source: BMC Endocr Disord. 2014 Jul 19;14:60. doi: 10.1186/1472-6823-14-60 (PMC4107728; doi:10.1186/1472-6823-14-60)
Supplement: Additional file 2: Table S2 — Registry of the Bibliographic Searches. [file 1472-6823-14-60-S2.docx]

Supplemental Table S2. Registry of the Bibliographic Searches

| **Databases** | **Platform/ Access** | **Search date** | **References retrieved** |
| --- | --- | --- | --- |
| **Core databases** | |  |  |
| EMBASE | Ovid Licensed Resource | 27/10/2012 | 770 |
| Medline | Ovid Licensed Resource | 27/10/2012 | 689 |
| CINAHL | EBSCO Licensed Resource | 27/10/2012 | 116 |
| Current Contents | ISI Licensed Resource | 27/10/2012 | 355 |
| CRD Databases (DARE, HTA) | [http://www.crd.york.ac.uk/crdweb/](http://nhscrd.york.ac.uk/) | 27/10/2012 | 6 |
| **Coverage/regulatory/licensing agencies** | |  |  |
| U.S. Centers for Medicare & Medicaid (CMS) Web site | [http://www.cms.hhs.gov/default.asp?](http://www.coverageandpayment.com/) | 18/10/2012 | 0 |
| AETNA | <http://www.aetna.com/index.htm> | 18/10/2012 | 0 |
| National Guidelines Clearinghouse | [http://www.ngc.gov](http://www.ngc.gov/) | 18/10/2012 | 0 |
| Mc Master Health Forum | <http://www.mcmasterhealthforum.org/healthsystemsevidence-en> | 20/10/2012 | 3 |
| **Grey literature** | |  |  |
| Networked Digital Library of Theses and Dissertations - electronic theses and dissertations (ETDs) | <http://www.ndltd.org/> | 20/10/2012 | 0 |
| **Other Internet Directories or search resources** | | 20/10/2012 |  |
| Health Evidence | <http://www.evidence.nhs.uk/> | 20/10/2012 | 5 |
| SCIRUS | http://www.scirus.com/ | 27/10/2012 | 380 |
| LILACS - Literatura Latinoamericana y del Caribe en Ciencias de la Salud | <http://bases.bireme.br/cgi-bin/wxislind.exe/iah/online/?IsisScript=iah/iah.xis&base=LILACS&lang=e> | 25/10/2012 | 0 |
| CEA Registry | <https://research.tufts-nemc.org/cear/default.aspx> | 25/10/2012 | 0 |
| metaRegister of Controlled Trials (mRCT) | http://www.controlled-trials.com/mrct/ | 25/10/2012 | 0 |
| Backward and forward search of the articles previously identified. ISI WEB OF KNOWLEDGE | <http://apps.webofknowledge.com/> | 30/11/2012 | 1275 |
| **TOTALREFERENCES SCREENED** |  |  | **3599** |
