# Supplementary material for: Characteristics and effectiveness of diabetes self-management educational programs targeted to racial/ethnic minority groups: a systematic review, meta-analysis and meta-regression
Source: BMC Endocr Disord. 2014 Jul 19;14:60. doi: 10.1186/1472-6823-14-60 (PMC4107728; doi:10.1186/1472-6823-14-60)
Supplement: Additional file 3: Table S3 — Characteristics and Effectiveness of the Diabetes Self-management Educational Interventions. [file 1472-6823-14-60-S3.docx]

| *Supplemental Table S3*. Characteristics and Effectiveness of the Diabetes Self-management Educational Interventions | | | | | | | | | | | |
| --- | --- | --- | --- | --- | --- | --- | --- | --- | --- | --- | --- |
| Setting | Target population | Duration of the intervention (months)/ Number of sessions | Educators | Delivery method | Teaching methods/ Educational contents | Design/ N/ Follow-up | Study Outcomes* | | | Overall quality | Study, Year (Reference), Country |
|  |  |  |  |  |  |  | Knowledge | Self-management activities | Clinical outcomes |  |  |
| Community Center | Mexican Americans, over 50 years old | 2 months/8 sessions | two  Local Mexican-American, bilingual, registered nurses with a  background in nutrition | Face to face + telecommunication/Group session/ Family not invited | D, SPS/  Dt, BDK | RCT/152 (Intervention=78, Control = 74)/  Follow-up  at 10 weeks and 14 weeks  post-intervention. | - | Kcal intake, cholesterol intake | Body weight | Poor | Elshaw et al, 1994 [43], US |
| Community Center | Mexican Americans | 2 months/ 9 sessions | Mexican-American clinical nurse specialist, dietitian, and community worker | Face-to-face/ Group sessions/ Family invited | D/  Dt, SMBG, BDK | non-controlled before-after study/ 5/ Follow up at 3 months post-intervention | **diabetes knowledge** | - | **fasting blood sugar levels, HbA1c,** cholesterol, triglycerides | Poor | Brown et al. 1995 [37], US |
| Hospital | Older African-American | 3 months/ 18 sessions | Dietitian | Face-to-face/ Group and individual sessions/ Family invited | D, SPS/  Dt, Ex | RCT/ 64 (Intervention= 32, Control=32)/ Follow up at completion and 3 months post-intervention | diabetes knowledge | Physical activity, nutrition, kilocalories intake | **Weight**, **BMI, Waist/hip ratio, HbA1c, Blood pressure**, Cholesterol, Triglycerides | Good | Agurs-Collins et al, 1997[27], US |
| General Practice | British Pakistani | 6 months/ Not reported | Link worker | Face-to-face/ Individual sessions/ Family not invited | D/  Dt, SMBG, DBK | RCT/ 201 (Intervention= 112, Control= 89)/ Follow up at completion | **diabetes knowledge** | **self-management behavior**, attitudinal views | HbA1c | Poor | Hawthorne and Tomlinson, 1997 [44], UK |
| Unclear | Caribbean Latinos | 16 months /20 sessions |  | Face-to-face/ Group sessions/ Family not invited | D, SPS/  Dt, Psy | RCT/ 38 (Intervention= 18, Control=20)/ Follow up at completion | - | **Nutrient intake** | - | Poor | Vazquez et at, 1998 [61], US |
| Home | African-American | 6 months/ 4 sessions | Health educator | Telecommunication/ Individual sessions/ Family not invited | D, SPS/  Psy | RCT/ 208 (Intervention= 137, Control= 143)/ Follow up at completion | - | **rate of ophthalmic examination** | - | Fair | Basch et al, 1999 [35], US |
| General Practice | South Asians from Surinam | 3 months /5 sessions | Nurse and dietitian | Face-to-face/ Individual sessions/ Family not invited | D/  Dt | RCT/ 113 (Intervention= 53, Control=60)/ Follow up at completion | - | - | HbA1c, **lipid profile,** BMI | Fair | Middelkoop et al, 2001 [51], Netherlands |
| Community Center | Mexican Americans | 12 months/ 52 sessions | Bilingual Mexican American nurses, dietitians, and community workers | Face-to-face/ Group sessions/ Family invited | D, SPS/  Dt, Ex, SMBG | RCT/ 252 (Intervention= 126, Control= 216)/ Post-intervention at 3, 6 and 12 months | **Diabetes Knowledge,** health believes | - | **HbA1c, fasting glucose,** cholesterol, triglycerides, BMI | Fair | Brown et al, 2002 [38], US |
| General Practice | Rural African Americans | Not reported/9 sessions | dietician and nurse case managers | Face-to-face/ Group sessions/ Family invited | D, SPS/  Dt | Non-controlled before-after study / 23/ Follow up at completion | - | **Fat related dietary habits, frequency of acute care visits** | **HbA1c, fasting blood glucose**, lipids | Poor | Anderson-Loftin et al, 2002 [31], US |
| Fitness center | Low-income, low-education, African American Women | 3 months/ 36 sessions | Dietician, nurses and diabetes educators | Face-to-face/ Group sessions/ Family not invited | D, SPS/  Dt, Ex, SMBG, BDK, Psy | Non-controlled before-after study /30/ Follow up at completion | **nutrition knowledge** | - | **total cholesterol and LDL-cholesterol levels, cardiovascular fitness, muscular strength and endurance,**  HDL, HbA1c, Fasting glucose, triglycerides, body weight | Poor | Rimmer et al, 2002 [53], US |
| General Practice | African-American women | 12 months/ 4 sessions | Community health workers, nutritionist | Face-to-face/ Individual and group sessions/ Family not invited | D, GSN , GSN/  Dt, Ex, SMBG | RCT/ 200 (Intervention A= 67, Intervention B= 67, Control= 66)/ Follow up at completion | - | **Physical activity** | - | Good | Keyserling et al, 2002 [45], US |
| Community Center | African American women | 2 months /6 sessions | Nurses, Community Health Worker and dietician | Face-to-face/ Group sessions/ Family not invited | D, GSD, SPS, CR/  Dt, SMGB, DBK | Non-controlled before-after study /25/ Follow up at completion | diabetes knowledge, self-efficacy | **diabetes-related emotional distress** | **HbA1c, weight, BMI** | Poor | Melkus et al, 2004 [50], US |
| General Practice | Ethnically diverse, medically underserved patients | 12 months/ 4 sessions | Nutritionists | Face-to-face/ Group and individual sessions/ Family not invited | D, GSD/  Dt, Ex, SMBG, Psy | RTC/  98 (Intervention=49, Control=49).  Follow-up at 3, 6 and 12 months. | - | - | weight, BMI, **HbA1c**, total cholesterol, LDL cholesterol, HDL cholesterol, triglyceride, **blood pressure** | Fair | Mayer-Davis et al, 2004 [49], US |
| General Practice | Ethnically diverse diabetic patients | 12 months/ 5 sessions | Diabetes educators and dietician | Face-to-face/ Group and individual sessions/ Family invited | D, GSD/  Dt, Ex, SMBG, Ma, Psy | Non-controlled before-after study/ 70/ Follow up at completion | - | - | BMI, **HbA1c** | Fair | Banister et al, 2004 [33], US |
| Community Center | low-income Spanish-speaking | 3 months/ 10 sessions | nutritionist, nurse, and intervention assistant (all bilingual) | Face-to-face/ Group and individual sessions/ Family invited | D, GSN/  Dt, Ex, SMBG, BDK, Ma, Psy | RCT/ 25 (Intervention=15, Control=10)/ Follow up at completion and 3 months post-intervention | - | **Dietary quality, reduction of total calories, percentage of fat of total calories, percentage of saturated fat of total calories, blood glucose self-monitoring,** physical activity, medication intensity | HbA1c, lipids, blood pressure, weight, waist circumference | Poor | Rosal et al, 2005 [55], US |
| Community Center | African-American | 2 months/ 6 sessions | Nurse and dietitian | Face-to-face + telecommunication/ Group and individual sessions/ Family not invited | D, SPS/ Psy | RCT/ 244 (Intervention=125, Control=114)/ Follow up at completion | Perceived understanding of diabetes, Diabetes empowerment scale, Attitude toward seriousness of diabetes | - | HbA1C , serum cholesterol, weight, blood pressure | Fair | Anderson et al, 2005 [29], US |
| General Practice | African Americans | 6 months/ 48 sessions | Nurse and dietitian | Face-to-face + telecommunication/ Group sessions/ Family not invited | D, SPS/  Dt | RTC/ 97 (intervention=49, control=48)  Follow-up at completion | - | **Dietary behaviors** | HbA1c, **BMI**, triglycerides, cholesterol | Fair | Anderson-Loftin, 2005 [30], US |
| Community Center | Mexican Americans | 12 months/ 8 sessions | Nurses,  dietitians, and community workers | Face-to-face/ Group sessions/ Family invited | D, SPS, CR/  Dt, Ex, SMBG | Non-controlled before-after study/102/ Follow-up at three months and at completion | **diabetes knowledge** | - | **Hb1c, Fasting blood glucose** | Good | Brown et al, 2005 [37], US |
| Community Center | Alaskan Eskimos | 48/1 | Not reported | Face-to-face/ Individual sessions/ Family not invited | D, SPS/  Dt | Controlled before-after study/664 (Intervention=388, Control=276)/ Follow up at completion | - | - | **total cholesterol, LDL cholesterol, fasting glucose, diastolic blood pressure, improved glucose tolerance,** weight | Poor | Ebbesson et al, 2005 [42], US |
| Community Center | African American and Latinos | 5 months/ 5 sessions | Family Health Advocate | Face-to-face/ Group sessions/ Family invited | D/  Dt, Ex, SMBG, BDK, Psy | Non-controlled before-after study/ 111/ Follow up at completion | **self-management knowledge** | Diet, physical activity, **self-care behaviors** | **HbA1c** | Poor | Two Feathers et al, 2005 [60], US |
| General Practice | Multiple ethnic minority groups | 3 months/ 3 sessions |  | Face-to-face/ Group sessions/ Family not invited | D, CR/  Dt, Ex, SMBG, DK | RCT/ 188 (Intervention=59, Control=59)/ Follow up at completion | **diabetes knowledge** | Attitudes towards seriousness | - | Poor | Baradaran et al, 2006 [34], UK |
| General Practice | Hispanic Americans | 2 months/ 6 sessions | Research team | Face-to-face/ Group sessions/ Family not invited | D, SPS/ Dt, Ex, SMBG, BDK, Ma, Psy | Non-controlled before-after study/16/ Follow up at 3 months post-baseline and at completion | **diabetes knowledge** | - | **lipid profiles, HbA1c** | Poor | Mauldon et al, 2006 [48], US |
| Community Center | Mexican American | 6 months/ 8 sessions | Team of nurses, dieticians, social workers, and community  health workers | Face-to-face and telecommunication/ Group sessions/ Family not invited | D, SPS/  Dt, SMBG, BDK, Psy | RCT/ 149 (Intervention=75, Control=74)/ Follow up at 3 months post-baseline and at completion | Diabetes health beliefs, **diabetes** **knowledge** | - | **HbA1c** | Good | Lujan et al, 2007[47], US |
| General Practice | Uninsured Mexican Americans | 12 months/ 7sessions | Community  health worker | Face-to-face/ Individual sessions/ Family not invited | D, GSN, CR/  Dt, Ex, SMBG, DK | Non-controlled before-after study/162/ Follow up at 6 months post-baseline and at completion | - | - | **Hba1c**, BMI | Poor | Culica et al, 2007 [40], US |
| General Practice | Mexican American | 2 months/ 8 sessions | Lay peer educators, and bilingual nurses and dieticians | Face-to-face/ Group sessions/ Family invited | D, GSN/  Dt, Ex, SMBG, BDK, Ma | RCT/ 17 (Intervention=9, Control=8)/ Follow up at completion and 4 weeks post-intervention | **diabetes knowledge** | self-efficacy, and self-care measures | **weight, BMI,** HbA1C, and fasting glucose | Fair | Vincent et al, 2007 [62], US |
| General Practice | African American women | 1 months/ 4 sessions | African  American geriatric nurse practitioner | Telecommunication/ Individual sessions/ Family not invited | D, SPS/  Dt, EX, SMBG, Ma, Spy | RCT/ 68 (Intervention=32, Control=36)/ Follow up at completion | - | **Exercise, psychosocial adjustment,** diet, blood sugar testing, foot care, medications | - | Fair | Amoako et al, 2008 [28], US |
| General Practice | Mexican American | 1 day/ 1 session | Diabetes educator | Face-to-face/ Individual sessions/ Family not invited | SPS, CR/  BDK | RCT/ 112 (Intervention=55, Control= 57)/ Follow up at 1 month post-intervention | - | **foot self-care behaviors** | - | Fair | Borges et al, 2008 [36], US |
| General Practice | Multiple ethnic minority groups | 9 months /9 sessions | Physician and health educator | Face-to-face/ Group sessions/ Family not invited | D, SPS/  Dt, Ex, SMBG, Ma, Spy | RCT/ 227 (Intervention=113, Control= 114)/ Follow up at completion |  | **self-management behavior** | HbA1c | Fair | Schillinger et al, 2009 [57], US |
| Home | Older African American Women | 9 months/ 4 sessions | Nurses | Face-to-face/ Individual sessions/ Family not invited | D/  BDK | RCT/ 180 (Intervention=120, Control= 60)/ Follow up at 3 and 6 months post-baseline and at completion | - | self-care activities | HbA1c | Fair | Skelly et al, 2009 [58], US |
| Clinic | Hispanic | 6 months /11 sessions | Community Health Worker | Face to face + telecommunication/ Individual sessions/ Family invited | GSN, SPS/ Dt, Ex, SMBG, Ma, Spy | RCT/ 129 (Intervention=75 , Control=54)/ Follow up at completion | **diabetes knowledge** | **Dietary habits, physical activity, medication adherence,** | HbA1c, BMI | Fair | Babamoto et al, 2009 [32], US |
| Community Center | Multiple ethnic minority groups from rural communities | 12 months /13 sessions | Dietitian and nurse/certified diabetes educator | Telecommunication/ Individual sessions/ Family not invited | GSD/  Dt, Ex, BDK, Ma, Psy | RTC/168 (intervention =85, control =80)/ Follow-up at 6 months and at completion | - | - | **HbA1c, LDL cholesterol,** blood pressure, BMI, Waist circumference, Albumin-to-creatinine ratio | Good | Davis et al, 2010 [41], US |
| General Practice | African-American and Latinos | 6 months/ 4 sessions | Medical Assistant Coach | Face to face + telecommunication/ Individual + group sessions/ Family not invited | D, GSN/  Dt, Ex, SMBG | RCT/ 75 (Intervention=50, Control=25)/ Follow-up at completion | Diabetes Empowerment, Problems with Diabetes | - | HbA1c | Good | Ruggiero et al, 2010 [56], US |
| Community Center | Latinos | 12 months/ 20 sessions | Nutritionist, Health Educator and lay assistant | Face-to-face/ Individual sessions/ Family invited | D, GSD, SPS, CR/  D, Ex, SMBG, BDK, Ma, Psy | RCT/ 252 (Intervention= 124, Control=128). Follow up at 4 months post-baseline and at completion. | - | **Reduction of total calories, percentage of fat of total calories, percentage of saturated fat of total calories, blood glucose self-monitoring,** physical activity, medication intensity | HbA1c, lipids, blood pressure, weight, waist circumference | Good | Rosal et al, 2011[54], US |
| Community Center | Mexican American | 4 months/ 8 sessions | Peer educator | Face-to-face/ Group and individual sessions/ Family not invited | D, SPS/  D, Ex, SMBG, BDK, Ma, Psy | RCT/ 207 (Intervention= 104, Control=103)/ Follow up at 4 months post-baseline and at completion. | - | - | **HbA1c, blood pressure**, cholesterol, triglycerides, BMI | Fair | Philis-Tsimikas et al, 2011[52], US |
| Community Center | Latinas | 24 Months/ 45 sessions | bilingual clinical staff and community professionals | Face-to-face/ Group sessions/ Family not invited | D/  Dt, Ex Psy | RCT/ 280 (Intervention=142, Control=138)/ Follow up at 6 and 12 months post-baseline and at completion. | - | Quality of problem solving strategies, Self-efficacy, **% Calories saturated fat, Physical activity, Chronic illness resources** | **BMI, HbA1c, CHD risk** | Fair | Toobert et al, 2011 [59], US |
| Home | Urban adults | 12months/10 sessions | Health Educator | Telecommunication/ Group sessions/ Family not invited | GSD, GSN, SPS/ Dt, Ex, Ma | RCT/ 526/ (Intervention=262, Control= 264)/ Follow up at completion | - | - | **HbA1c** | Fair | Walker et al, 2011 [63], US |
| Home | African American veterans | 6 months/ Not reported | Peer mentor | Telecommunication/ Individual sessions/ family not invited | GSN, CR/ Psy | RCT/ 78 (Intervention=39, Control=39)/ Follow up at completion | - | - | **HbA1c** | Good | Long et al, 2012 [46], US |

* Study outcomes in bold represent those that showed a statistically significant improvement after the intervention. BMI, Body Mass Index; HbA1c, Glycated hemoglobin; CHD, cardio heart disease; D, Didactic; SPS, Situational problem solving; GSN, Goal setting negotiated; GSD, Goal Setting Dictated; CR, Cognitive reframing, Dt, diet; BDK, Basic diabetes knowledge; SMBG, self-monitoring blood glucose; Ex, exercise; Psy, Psychosocial; Ma, Medication Adherence
